# Inflammasome/IL-1β Responses to Streptococcal Pathogens

**DOI:** 10.3389/fimmu.2015.00518

**Published:** 2015-10-08

**Authors:** Christopher N. LaRock, Victor Nizet

**Affiliations:** ^1^Department of Pediatrics, University of California San Diego, La Jolla, CA, USA; ^2^Skaggs School of Medicine and Pharmaceutical Sciences, University of California San Diego, La Jolla, CA, USA

**Keywords:** inflammasome, caspase-1, IL-1β, pyroptosis, *Streptococcus*

## Abstract

Inflammation mediated by the inflammasome and the cytokine IL-1β are some of the earliest and most important alarms to infection. These pathways are responsive to the virulence factors that pathogens use to subvert immune processes, and thus are typically activated only by microbes with potential to cause severe disease. Among the most serious human infections are those caused by the pathogenic streptococci, in part because these species numerous strategies for immune evasion. Since the virulence factor armament of each pathogen is unique, the role of IL-1β and the pathways leading to its activation varies for each infection. This review summarizes the role of IL-1β during infections caused by streptococcal pathogens, with emphasis on emergent mechanisms and concepts countering paradigms determined for other organisms.

## Introduction

Humans are frequently colonized by pathogenic species of streptococcal bacteria: the throat and skin by *Streptococcus pyogenes* (group A *Streptococcus*; GAS), the upper respiratory tract by *Streptococcus pneumoniae* (pneumococcus, SPN), and the lower intestine and genital tract by *Streptococcus agalactiae* (group B *Streptococcus*; GBS). This microbial–host association usually occurs in the context of asymptomatic colonization or superficial mucosal infection, but each of these pathogens can also be associated with severe, invasive, even life-threatening, diseases. GAS causes a wide range of diseases, including pharyngitis, cellulitis, puerperal sepsis, necrotizing fasciitis, streptococcal toxic shock syndrome, and rheumatic heart disease, making it one of the top 10 causes of infectious mortality (1). SPN is a similarly prevalent human pathogen responsible for greater than one million annual deaths by pneumonia and meningitis, mostly in young children (2). Lastly, GBS is a common cause of neonatal sepsis and meningitis, making it an important cause of infectious morbidity and mortality among infants in many countries throughout the world (3).

Inflammation is a key component of the immune response during infections with all of the pathogenic streptococci. Inflammation can be protective by preventing bacterial colonization, replication, invasion, and dissemination. Insufficient inflammation commonly leads to a greater infection susceptibly or more prolonged disease. Conversely, excessive inflammation is a driver of several autoimmune diseases and of host tissue injury complicating many severe infectious diseases. Inflammation must therefore be carefully regulated for an optimal immune response, and pathogens can exploit the regulatory processes deployed by the host innate immune system. For example, inflammation in the upper respiratory tract increases the risk of systemic dissemination of SPN, even though it is critical for combating the localized infection at that site. For SPN as well as GBS, inflammation helps break down the blood–brain barrier (BBB) to cause meningitis. In these deadly infections, the tissue damage resulting from inflammation can lead to acute complications, and even if the pathogen is successfully cleared, can be associated with post-infectious sequelae.

The IL-1 and inflammasome pathways in particular exemplify the complex role of inflammation during streptococcal infection. Indeed, GAS is classically defined as a “pyogenic” pathogen, exemplified by pus formation elicited by the robust inflammatory response to its tissue invasion. IL-1β is a highly inflammatory cytokine commonly key in eliciting protective immunity. Caspase-1 and its canonical regulator the inflammasome were first discovered for their ability to activate IL-1β. The inflammasome pathway has since been found to regulate numerous other inflammatory and antimicrobial activities, which in several instances contribute more to the functional immunity than does IL-1β. Activation of IL-1β is also not fully dependent on the inflammasome, but instead requires cooperation between several pathways, many of which also can be activated along redundant routes. When distinctions can be made based on the available literature, we attempt to disambiguate the contribution of each of these signaling and immune effector pathways.

## Biology of IL-1β and the Inflammasome

### IL-1

The IL-1 receptor (IL-1R) is widely expressed, which allows IL-1 signaling to induce a variety of cellular effector mechanisms locally as well as systemically. Two cytokines, IL-1α and IL-1β, are recognized by IL-1R to similar effects. The major distinction between these cytokines is that IL-1β is soluble, while IL-1α is typically membrane bound, spatially limiting its function to the activation of neighboring cells. By contrast, IL-1β is free to also act as a chemokine and mediate systemic signaling events. IL-1R1^−/−^ mice, deficient for cell signaling in response to both IL-1α and IL-1β, are more susceptible to most infections, including those caused by GAS (4), GBS (5), and SPN (6–9).

IL-1α is a key mediator of the sterile inflammatory response (10), but is not generally critical for the response to bacterial infection (11). Nevertheless, IL-1α is stimulated during infections by SPN (12, 13), GBS (14), and GAS (15). Genome-wide linkage studies in mice identified a correlation between IL-1α levels and mortality during GAS sepsis (15), suggestive that IL-1α contributes to cytokine storm during sepsis. However, this link was not found in human studies focused on skin infections (16), perhaps because IL-1α might be more beneficial than detrimental in this context. IL-1α probably plays at most a minor role during streptococcal infections, as IL-1β^−/−^ mice phenocopy IL-1R^−/−^ mice in their resistance to GBS (5, 17). The role of IL-1α during experimental GAS and SPN infections is not yet clear.

IL-1β is critical in defense against GAS (4, 18), GBS (19), and SPN (6, 9, 20, 21). IL-1β is a major chemoattractant of neutrophils (10), and neutrophil recruitment is largely mediated by IL-1β during GAS (4) and GBS infections (17). This neutrophil influx to the site of infection contributes to GAS and GBS killing, since neutrophil ablated and IL-1R^−/−^ mice have a similar susceptibility to these pathogens (4, 17). SPN is largely resistant to recruited neutrophils during pneumonia, but rather succumbs to the wave of activated macrophage that follows, which is also largely IL-1β dependent (6, 9, 20, 22). IL-1β also induces fibrinogen expression and localized coagulation, which help to limit dissemination of SPN from the lung (8). It is not clear if this occurs during other streptococcal infections, but if so, the effects may not always benefit the host, as both GAS and GBS have surface-expressed virulence factors that bind fibrinogen and interfere with complement activation and phagocytosis (1, 3).

By controlling early bacterial infection before it becomes this severe, IL-1 can help prevent a pathogen from reaching immune-privileged or vulnerable sites, such as the central nervous system (CNS). Consistent with this notion, IL-1 signaling-deficient mice develop meningitis as a complication of respiratory tract infections at a higher frequency (7). However, once a pathogen reaches the BBB, inflammation is often more harmful then beneficial. GBS crosses the brain microvascular endothelial cells comprising the BBB by direct intracellular invasion (23) without inducing IL-1 (24). SPN can similarly invade the cerebral endothelial cells to gain access to the CNS without barrier damage or disruption (25). Despite these non-inflammatory mechanisms for gaining CNS entry, bacterial CNS infections are inherently inflammatory. Bacterial growth and damage to the initially infected CNS cells greatly induces IL-1 (26), which further breakdowns the BBB to allow more bacterial invasion (27). IL-1 also recruits and activates neutrophils, which are overtly injurious in murine meningitis models (28, 29) and may correlate with poor patient prognosis (30). Neutrophils in the CNS are ineffectual against SPN (31), so there is unfortunately little obvious benefit to this inflammation. Moreover, IL-1 contributes to the pathogenesis of numerous neurodegenerative diseases, and likely has direct role in neurological sequelae common among survivors of streptococcal CNS infection (2, 3, 30, 32).

### Interleukin-18

Interleukin-18 (IL-18) is another inflammasome-regulated proinflammatory cytokine. The largest contribution of IL-18 to immunity lies in stimulation of natural killer (NK) cells and induction of interferon-γ (IFN-γ) signaling (33, 34). IL-18 activation is seen during GAS (18, 35), GBS (19), and SPN infections (36). IL-18^−/−^ mice are more susceptible to SPN pneumonia (37). However, in a SPN meningitis model, IL-18^−/−^ mice actually survived longer than WT controls, suggesting that inflammation induced by IL-18 may be more pathological than beneficial in CNS infection, as is the case for IL-1 (38). In GBS infection of neonatal mice, an IL-18 neutralizing antibody increased GBS burden and mortality; conversely, administration of recombinant IL-18 reduced GBS counts (39).

### Pyroptosis

**Figure 1:**
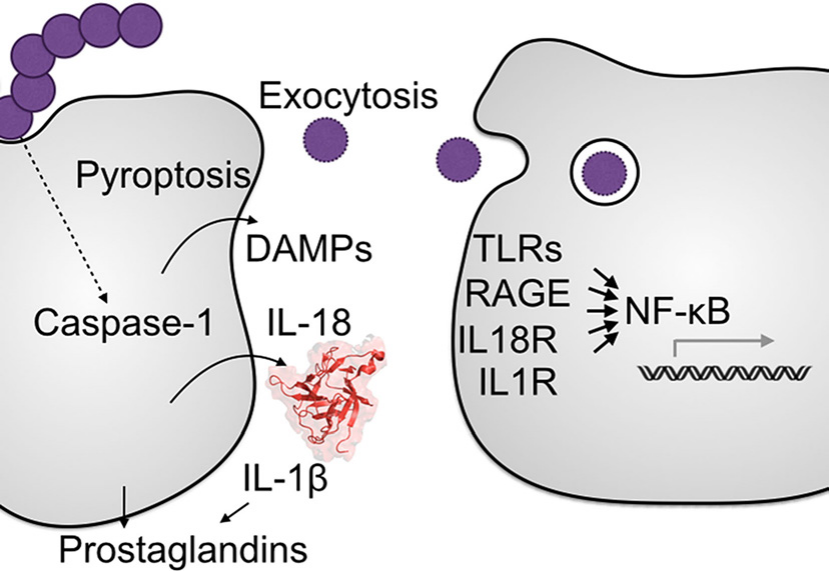
**Major effector mechanisms of the inflammasome**. A cell containing active inflammasomes releases several inflammatory signals to other cells, including prostaglandins/eicosanoids, IL-1β, and IL-18. The other major cell process activated is programed cell death by pyroptosis, whereupon the released cellular contents can be detected by a number of pattern-recognition receptors to further inflammatory signaling. Pyroptotic cell death also releases any intracellular pathogens, exposing them to direct killing by complement or antimicrobial peptides or phagocytosis by neighboring cells.

In addition to cytokine signaling, activation of inflammasomes initiates programed cell death by pyroptosis (Figure 1). This form of cell death releases numerous endogenous damage-associated molecular patterns (DAMPs), including ATP, DNA, HMGB1, and histones, which further amply the inflammatory response through the recruitment and activation of neutrophils and other immune cells (34). Due to the abundance of DAMPs released during pyroptosis, much of the inflammasome-driven inflammatory response during infection can progress in an IL-1- and IL-18-independent manner (11). In the instance of pneumococcal meningitis, neutralization of IL-1 and IL-18 ameliorate a remarkable amount of the inflammation, yet not all of it (29). A DAMP released during pyroptosis that strongly induces inflammation is HMGB1, a chromatin protein recognized by TLR4 and RAGE receptors. Extracellular HMGB1 is abundant during SPN meningitis, with the levels correlating to severity of disease in both mice and humans (40).

In addition to inflammation, pyroptotic cell death plays an important role in immunity by depriving intracellular pathogens of a replicative niche. Intracellular bacteria are protected from many innate and cellular immune defenses; lysis releases the bacteria where they are exposed to immune cells that are primed and better able to combat the pathogen (41). Though they are commonly treated as exclusively extracellular pathogens, the streptococci can specifically remodel the cellular antimicrobial response to allow intracellular replication (42, 43). It is not yet clear how protective pyroptosis might be for the host during streptococcal infection, but GAS is able to use it to its own advantage. Compared to other cell death programs, pyroptosis occurs relatively rapidly. GAS induction of cell death can be so rapid that IL-1 production is limited, since the cell does not have time to synthesize and convert much cytokine (44).

### Other Mechanisms

Several emergent inflammasome effector pathways may also play a role in combating streptococcal infection. The inflammasome can induce secretion of prostaglandin E2, both directly and through IL-1β-induced cell signaling (45). Prostaglandin E2 is markedly induced during GAS (46), GBS (47), and SPN infection (48). This induction has been observed in several infection models including sepsis (15), necrotizing fasciitis (49), and puerperal infection (50). *In vitro*, prostaglandin E2 is immunosuppressive and impairs killing of GAS (49) due to repression of phagocytosis, reactive oxygen species, and inflammatory cytokines like TNF-α (50). Consistent with these observations, COX-2^−/−^ mice, deficient in prostaglandin E2, had greater GAS resistance (49). However, COX-2-targeting non-steroidal antiinflammatory drugs have long been thought to exacerbate GAS infection and be a risk factor for developing invasive infections (51); therefore, the role of prostaglandin E2 in the anti-GAS immune response is not entirely clear.

Inflammasome activation might also act against intracellular bacteria by mechanisms that do not require death of the host cell. Caspase-1 promotes greater acidification of the phagolysome in GBS-infected cells (52). This mechanism appears to be inactive during infections with Gram-negative bacteria, but operates in response to the Gram-positive bacteria tested, so would likely act against GAS and SPN as well. IL-1β signaling provides another route for killing of several species of intracellular bacteria, including GAS (18). This effect is mediated through autocrine induction of IL-1R-regulated pathways, but which antimicrobial effectors are ultimately involved is not yet known.

## The Inflammasomes

### Caspase-1

The inflammasome is a scaffold nucleotide-binding domain and leucine-rich repeat containing receptor (NLR) family of proteins that serves to activate a component conserved between inflammasomes: the cysteine protease Caspase-1. Caspase-1^−/−^ and IL-1β^−/−^IL-18^−/−^ mice often exhibit similar infection response phenotypes (11). The immune contributions of pyroptosis and other cytokine-independent inflammasome effector mechanisms can make the role of Caspase-1 more prominent in certain infections. Alternatively, inflammasome-independent mechanisms for IL-1β secretion can shift this balance in the other direction (34). Consistent with inflammasomes playing a protective role during streptococcal infection, Capase-1^−/−^ mice are more susceptible to GAS (18) and GBS (19). The importance of Caspase-1 in defense against SPN varies greatly depending on model, mirroring the variable role of IL-1 in these infections. In a SPN pneumonia model, Caspase-1 had little effect (18, 53), but in a SPN meningitis model, Capase-1-driven inflammation led to great intracranial pressure and disruption of the BBB (26).

### NLRP3 Detection of Pore-Forming Toxins

**Figure 2:**
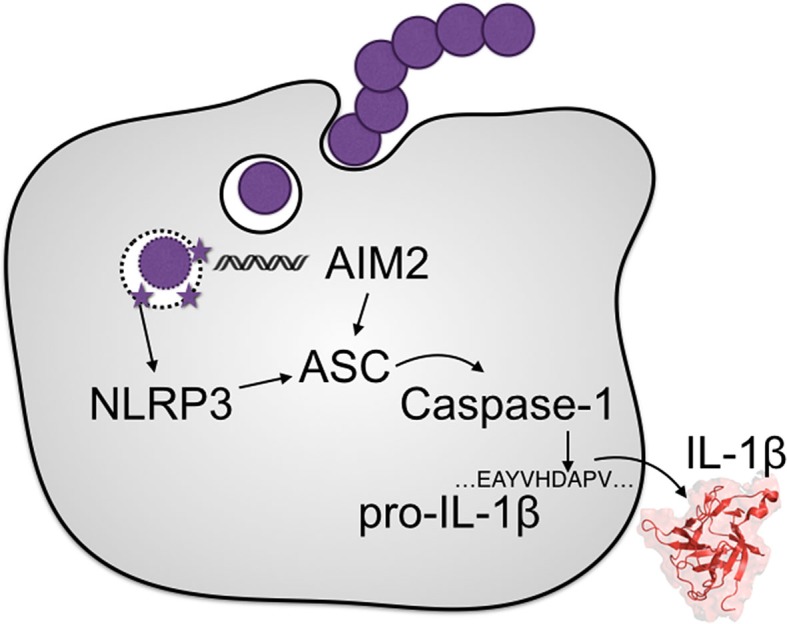
**Inflammasome activation by the streptococci**. Two primary NLRs form inflammasomes during streptococcal infections, NLRP3 and AIM2. NLRP3 detects membrane disruption by the pore-forming toxins encoded by all the major streptococcal species. These pores also allow bacterial DNA in to the cytosol for detection by AIM2. Either NLR can form an inflammasome scaffold for the activation of caspase-1, the primary protease responsible for the hydrolysis of IL-1β into its mature form.

Several different NLRs can form inflammasomes, but NLRP3 has the most prominent contribution for detection of streptococci (Figure 2). Streptococcal pathogens deploy secreted pore-forming toxins, which are well documented to activate the NLRP3 inflammasome (13, 19, 21, 29, 30, 54–57). The precise mechanisms by which NLRP3 senses diverse toxins from a number of bacterial species, as well as numerous other PAMPs-like crystals of uric acid, cholesterol, or amyloid proteins, is not entirely clear. Given the disparate nature of these molecules, and no known binding interactions, NLRP3 does not appear to directly detect these PAMPs and DAMPs. Several models have been put forward describing a mechanism for NLRP3 activation in response to perturbations in cellular homeostasis. This concept requires a secondary molecule commonly altered by these PAMPs and DAMPs. While the identity of this molecule is not agreed upon, a unifying theme is the disruption of either the outer membrane or endosomal membranes and consequent induction of ER stress (58). As not all NLRP3 stimuli are membrane acting, upstream detection pathways may still be involved in some circumstances. Streptococcal pore-forming toxins directly induce membrane disruption and ER stress (59), so their detection will likely follow whatever paradigm emerges to integrate the different models of NLRP3 activation.

Major pore-forming toxins of GAS and SPN are the cholesterol-dependent cytolysins streptolysin O (SLO) and pneumolysin (PLY), which use cholesterol and glycans as cell surface receptors (60). Both toxins form very large pores in many cell types. In immune cells, pore formation contributes to virulence by killing the cell or inactivating its effector mechanisms, but concurrently activates IL-1β secretion through the NLRP3 inflammasome (12, 13, 44, 54, 61, 62). GAS expresses a second membrane-active pore-forming toxin, streptolysin S (SLS), which is responsible for the classical β-hemolytic phenotype of GAS (63, 64). SLS does not contribute strongly to NLRP3 inflammasome activation (44). This may be due to a dominant role of SLO or the less potent lytic activity of SLS against non-erythrocytes (65), though a toxin’s ability to form pores and to activate the inflammasome do not always correlate strictly (62).

The major pore-forming toxin of GBS, β-hemolysin, is highly dissimilar to PLY, SLO, and SLS. This toxin stays tightly associated with the cell surface and plays a key role in the progression from colonization to invasive infection (66). β-hemolysin mutant GBS induce less pyroptosis and IL-1β secretion through the NLRP3 inflammasome (19). The mutation involved, *cylE*, also disrupts synthesis of the characteristic pigment of GBS granadaene (67). Granadaene itself is sufficient to activate the NLRP3 inflammasome (56), and production of granadaene is also linked to the hemolytic activity of *Propionibacterium jensenii* (68). While suggestive that granadaene is itself the β-hemolysin, CylE expression in *Escherichia coli* confers hemolytic activity but not pigmentation (67), and certain media conditions induce GBS pigmentation without a commensurate increase in hemolytic activity (69). An additional GBS toxin, CAMP factor, also forms pores and delivers bacterial products into the cytosol (70). While this activates several immune detection pathways, the inflammasome does not appear to be one of them for unknown reasons (19).

Pore-forming toxins also activate cell death processes that have features of osmotic lysis, apoptosis, necrosis, and oncosis, which can be confused for pyroptosis and complicate analysis of inflammasome activation (59, 71–74). Since maintaining cell membrane integrity is essential for viability and continued cytokine production, pore-forming toxins can, somewhat paradoxically, actually limit IL-1β by inducing these cell death pathways. The pore-forming toxins of GAS (44), GBS (75), and pneumococcus (74) each can induce the cell to lyse before much IL-1β can be synthesized and processed. Detection of pore-forming toxins, through both caspase-1-dependent and -independent pathways, can also induce membrane-healing mechanisms that limit toxin potency and cell death (34, 76). Therefore, the effect of toxins on the inflammasome appears to be highly concentration dependent: low doses promote cell activation and repair mechanisms, moderate doses activation of the NLRP3 inflammasome, and high doses a rapid cell death that limits IL-1β-driven inflammation.

### Alternative NLRP3 PAMPs

Some of the earliest results on the detection of pore-forming toxins by NLRP3 suggested that SLO is not sufficient for inflammasome activation (77). One explanation for this observation is that the NLRP3 inflammasome requires co-stimulatory signals for activation (78). Another explanation for this finding is that low concentrations of pore-forming toxin, themselves insufficient for inflammasome activation, can still mediate the delivery of inflammasome-activating PAMPs and DAMPs, such as bacterial RNA, CpG DNA, Pam_3_CSK_4_, zymosan, muramyl dipeptide, and lysozyme-digested peptidoglycan (13, 57, 79–81). Even in circumstances where toxin pore formation is sufficient for inflammasome activation, delivery of these additional PAMPs may provide for a stronger inflammasome stimulus and may allow activation of additional inflammasomes beyond the NLRP3.

Another GAS virulence factor, SpyA, can activate the NLRP3 inflammasome (18). SpyA is delivered in to host cells where it transfers ADP-ribose from nicotinamide adenine dinucleotide (NAD) onto host proteins to modify their activity (82). ADP-ribosylating toxins from *Pseudomonas aeruginosa* and *Mycoplasma pneumoniae* also activate the NLRP3 inflammasome (83), but the precise mechanism underlying the detection of these toxins is unclear. An ADP-ribosyltransferase toxin from *Clostridium botulinum* instead activates a pyrin inflammasome (84), suggesting the target of the toxin dictates which inflammasome is involved. Consistent with this hypothesis, other toxins that target Rho-GTPases like the Clostridial toxin are also detected via pyrin (84). One target of the *M. pneumoniae* toxin is NLRP3 (83), suggesting this could be a target of SpyA and other NLRP3 activating microbial enzymes. Alternatively, SpyA targets vimentin (85), which might de-repress the NLRP3 inflammasome (86). Additionally, ADP-ribosylating toxin depletion of NAD might activate the NLRP3 inflammasome (87); SpyA has very potent NAD-glycohydrolase activity (82). This suggests that another NAD-glycohydrolase of GAS, Nga can activate the inflammasome. Consistent with this hypothesis, Nga does induce cell death, but whether it is morphologically similar to pyroptosis and occurs through the inflammasome has not yet been determined (88).

### Alternative Inflammasome and IL-1β Pathways

A second inflammasome pathway activated during streptococcal infection proceeds through AIM2 in response to cytosolic double-stranded DNA from lysed bacteria (Figure 2). This PAMP is introduced into the cytosol upon the disruption of the phagosomal membrane by pore-forming toxins, such as PLY (89–91). The AIM2 inflammasome is important in the resistance to SPN (89, 91), but not GAS or GBS (19, 57). Since GAS and GBS are readily detected by other intracellular nucleic acid receptors (57, 70, 92–97), the mechanism underlying AIM2’s unresponsiveness is unclear.

The other well-studied inflammasomes, formed via NLRC4, NLRP1, or caspase-11, are not known to be involved in streptococcal infection. They have not been rigorously tested in the context of streptococcal infection, because streptococci do not possess PAMPs similar to those classically known to be detected by these receptors. NLRC4 is exclusively responsive to the flagellin and type III secretion rod proteins of Gram-negative bacteria (98), so expectedly, is unresponsive toward GAS (54). The best established PAMPs for the NLRP1 inflammasome are the *Bacillus anthracis* lethal toxin and an unknown factor of *Toxoplasma gondii* (99). Lastly, caspase-11 can form “non-canonical” inflammasome in response to the lipopolysaccharide of Gram-negative pathogens, but is felt to be non-responsive toward Gram-positive bacteria in general (98).

Group B *Streptococcus* and SPN similarly stimulate multiple pathways for inflammasome activation, and NLRP3^−/−^ mice are more susceptible to infection by these pathogens (19, 55, 89). However, there are very likely additional mechanisms allowing for IL-1β activation during streptococcal infection, either by alternative inflammasome or by inflammasome-independent mechanisms. The most telling evidence for this is that all the known inflammasome-activation PAMPs of GAS are detected by NLRP3 (18, 44), but NLRP3 does not contribute to resistance against GAS (54). IL-1β is nonetheless important in the immune response to GAS (4), but the source of its activation remains unclear.

The lack of a phenotype in NLRP3^−/−^ mice could be due to redundancy with AIM2, or with another, uncharacterized, inflammasome receptor that detects GAS. The NLR family of pattern-recognition receptors contains dozens of members with unassigned function, so many conventional inflammasomes may yet to be discovered. Alternatively, there may be inflammasome-independent pathways providing for IL-1β signaling. The GAS secreted protease SpeB cleaves and inactivates important immune factors such as immunoglobulins and antimicrobial peptides, making it important in several virulence models (1). In a biochemical assay, SpeB was found to cleave IL-1β (100). However, the pro-domain of IL-1β might just be intrinsically protease labile since it can also be cleaved by proteases from *Candida albicans* (101), *Entamoeba histolytica*, (102) *Staphylococcus aureus* (103), and *Treponema denticola* (104). *In vivo* activation of pro-IL-1β appears nevertheless to be quite specific, as caspase-11 is similar to caspase-1 and presumably cleaves some of the same substrates in order to activate pyroptosis, yet it does not process IL-1β (105). It further remains unclear whether cleavage by proteases other than caspase-1 can occur during infection, or whether it would promote or inhibit IL-1 signaling.

## Priming of the Inflammasome and IL-1

### Induction of IL-1 and the Inflammasome

At several points, the inflammasome and IL-1β signaling pathways intersect with the NF-κB pathway. First, most cells do not constitutively express IL-1β, which is transcriptionally regulated by NF-κB (106). Therefore, most TLR pattern-recognition receptors, acting through MyD88, as well as the subset of NOD receptors that signal through RIP2, can activate NF-κB to induce synthesis of pro-IL-1β (79). IL-1β will also positively regulate itself, since the IL-1R also activates NF-κB (106). Second, both the NLRP3 and AIM2 inflammasome require priming. This priming can occur through TLRs, IL-1R, or TNFR (36, 78, 107, 108). The AIM2 inflammasome is additionally primed by Type I IFN signaling (109), which simultaneously represses the NLRP3 inflammasome (110). GAS, GBS, and SPN can all induce IFN (70, 91–93, 111–113), which could therefore lead to switching of which inflammasomes can form, and consequently, which bacterial factors are detected.

**Figure 3:**
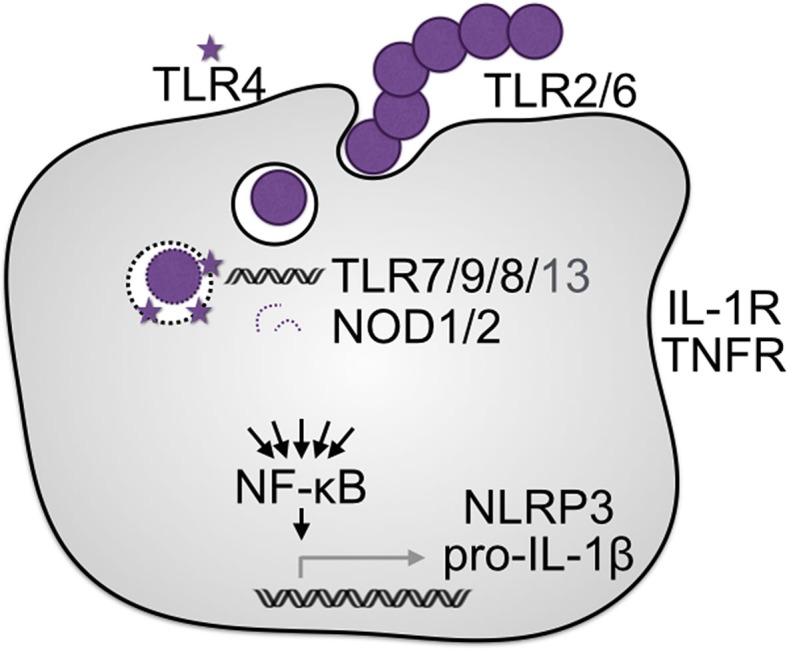
**IL-1β/inflammasome licensing pathways**. IL-1 and the NLR proteins responsive to the streptococci require induction. Cell–cell signaling can provide this priming signal in the form of IL-1β or TNF-α. More commonly during bacterial infection, bacterial factors detected as pathogen-associated molecular patterns by TLRs provide this signal. TLR2 detects bacterial lipoproteins and is broadly sensitive to Gram-positive pathogens. TLR4 is able to detect the pore-forming toxins of several species of streptococci. When the streptococci are intracellular and the phagosome is disrupted, several additional receptors are involved. TLRs 7, 8, 9, and 13 detect bacterial nucleic acids, while NOD1 and NOD2 detect bacterial cell wall fragments.

Since the NLRP3 and AIM2 inflammasomes are the only ones known to respond to streptococci (Figure 2), stimulatory pathways, such as TLRs, are critical not only for the induction of pro-IL-1β but also its maturation. We will therefore next discuss which of these pathways are known to detect streptococci, and how this detection promotes inflammasome/IL-1 signaling (Figure 3). Due to the large number of streptococcal PAMPs contributing to functional redundancies among TLRs, it might be expected that there would often be no immune susceptibility phenotype for any single TLR knockout (114). Nonetheless, through the use of streptococcal and host mutants several specific pathways have been identified. Of further note, a receptor may be found to be essential in one study and dispensable in another; when possible we note how streptococcal genotype, host genotype, and cell or infection model may impact these observations.

### Pattern-Recognition Receptor Detection of Streptococcal Pathogens

TLR2 activates NF-κB upon detection of bacterial lipopeptides, lipoteichoic acid, and peptidoglycan (115). These are ubiquitous cell surface components of Gram-positive bacteria, so TLR2 readily detects GAS (95, 116), GBS (117–120), and SPN (121–123). TLR6 and TLR1 cooperate with TLR2 to dictate which PAMPs stimulate signaling. TLR6 contribute to detection of GBS (118, 124) and SPN (53). For GAS, TLR6 is suggested to be dispensable, but through a dendritic cell model where TLR2 was also dispensable, in contrast to findings with other cell types (114). Even less is certain about TLR1, but it appears to have an overall lesser role upstream of inflammasome activation (118). GBS mutants unable to decorate their cell surface with lipoproteins induce less TLR2 signaling, but the contribution of any particular lipoprotein is unknown (120). The most abundant protein on the GAS surface, M protein, is also detected by TLR2 to stimulate production of several cytokines including IL-1β (125, 126). Lipoteichoic acids may also be detected by TLR2, though GBS lipoteichoic acid is not (115, 120). On possible explanation is that the streptococci post-translationally modify their lipoteichoic acid structure (127); however, since lipoproteins also commonly contaminate lipoteichoic acid preparations (115), this scientific question remains somewhat controversial.

TLR2 activation is specifically connected to the model of inflammasome licensing. Induction of *il1a*, *il1b* (9, 122, 128), and *nlrp3* (21) during SPN infection occurs through TLR2, which was required for normal levels of IL-1β signaling (55). TLR2^−/−^ mice are not as attenuated to in their cytokine responses to GBS or SPN infection as MyD88^−/−^ mice that are broadly deficient in TLR signaling (117, 128). This finding illustrates that while TLR2 is the canonical receptor for Gram-positive pathogens, additional receptors are activating NF-κB in parallel. Several TLRs more commonly appreciated for their role Gram-negative bacterial and viral infections have also been found to detect streptococci, suggesting their agonist range is broader than commonly appreciated.

TLR4 is the established receptor for lipopolysaccharide, a potent PAMP decorating the surface of Gram-negative bacteria, analogous to the broad importance of TLR2 for detection of Gram-positive bacteria. However, TLR4 is also able to detect PLY (129) through direct binding (130) independent of pore-forming activity (131). Consequently, TLR4 can compensate for TLR2 deficiency (122) to provide resistance to SPN pneumonia (123, 130, 132). PLY-deficient SPN induce less inflammasome-dependent cytokines IL-1α, IL-1β, and IL-18, with only a modest decrease in other cytokines such as TNF-α, IL-6, and IL-12 (12). The transcription of *il1b* is not greatly impacted by PLY (55), suggesting the toxin is more important for inducing NLRP3 than TLR4. This likely reflects a greater redundancy in the number of activating PAMPs for TLRs relative to NLRs leading to induction of their respective pathways (13). Nonetheless, TLR4 significantly potentiates caspase-dependent death induced by purified PLY (130). TLR4 detection of toxins may be a general mechanism since it has also been shown to mediate responses against several toxins including SLO from GAS (131). TLR4 is not important for detecting GBS (75), possibly due to TLR redundancy or because the GBS pore-forming β-hemolysin lacks homology with other pore-forming toxins (56, 67).

Several nucleic acid receptors are also known to recognize streptococci. ssRNA is recognized by TLR7 and contributes to the detection of GBS (92) but not GAS (93). Unmethylated bacterial DNA can be detected by TLR9, which leads to cell activation in response to SPN (53), GBS (92), and GAS (97). In one study, TLR7 and TLR9 were found to be much more important for the detection the detection of GAS and GBS than was TLR4 (92). For controlling SPN infection, TLR1, TLR2, TLR4, and TLR6 were functionally redundant but TLR9 was essential (53). In more recent studies, TLR7 and TLR9, as well as TLR2, TLR3, and TLR4, had minor roles in the detection of GAS and GBS compared to TLR8 (133). Like TLR7, TLR8 recognizes ssRNA, but this receptor is only present in humans, possibly leading to an overestimation of the relative importance of other TLRs in studies utilizing murine models. Mice instead express TLR13, not found in humans, which recognizes rRNA from several species including GAS (95). While some variation between studies is no doubt due to infection model differences, bacterial genetics can also be contributing variable. Hypervirulent M1T1 strains of GAS secrete a phage-encoded nuclease, Sda1, which degrades their own CpG-rich DNA to evade this detection by TLR9 (96). Similar mechanisms may allow the other streptococcal pathogens to evade TLR9, as well as other nucleic acid-sensing TLRs or NLRs.

NOD1 and NOD2 are related to NLRP3 and NLRC4 but activate NF-κB instead of the inflammasome. Both NOD proteins recognize muramyl dipeptide, a cleavage product of the peptidoglycan that comprises the bacterial cell wall (134) that can be introduced into the cytosol by pore-forming toxins (135). SPN is recognized by NOD1 (136) and NOD2 (137) through a process that requires PLY (136, 138) and bacterial cell wall degradation by lysozyme (81). Macrophages are the major cell recognizing SPN by NOD2 in a pneumonia model (138) with microglia and astrocytes-mediated detection during meningitis (139). NOD2 also is responsive to the GAS cell wall fragments, a commonly used inducer of inflammation in arthritis models (140). It is unknown whether NOD2 detects GAS during infection, and only a minimal role in GBS infection was detected (70, 75, 141). This result could be due to redundancy with other activation pathways since, even for SPN, NOD2 is largely redundant with TLR2 (138). Alternatively, streptococci might evade NOD detection through the same cell wall modifications that prevent detection by other PRRs and confer resistance to lysozyme (127).

### Integration of Additional Signaling Pathways

Several of the endogenous DAMPs released during pyroptosis may further amplify the local inflammatory response (10). This second phase of the response could provide for stimulation of TLRs that do not recognize the pathogen directly, which may be particularly important during infection with pathogens adept at evading TLR recognition. Given the multitude of TLR receptors identified to recognize streptococci and their components, pyroptosis might not be essential for initiating an immune response to these pathogens, but would nonetheless amplify inflammation during these infections. Pyroptotic release of DAMPs can also provide an alternative pathway to NF-κB activation in individuals with IRAK-4 deficiencies, who cannot signal via most TLRs with the exception of TLR3, and have an increased susceptibility to SPN and other pathogens (142).

## Conclusion and Perspective

A growing body of evidence suggests that there is more depth and complexity to IL-1β signaling than previously appreciated. For one, the inflammasome has been found to regulate several pathways in addition to IL-1β, including additional inflammatory signaling cascades, programed cell death, and antimicrobial effector mechanisms. Conversely, the number of pathways that can result in IL-1β activation is also increasing. As the inflammasome field grows, these new discoveries will provide greater insight on the molecular pathogenesis and host response to streptococcal infections. In a complementary fashion, experimental observations made using the streptococci and their unique suite of virulence mechanisms for altering the host response can help shape our understanding of the IL-1β/inflammasome pathway(s), which are so broadly impactful in clinical medicine.

How do alterations in the IL-1β/inflammasome response alter the incidence and outcome of streptococcal infections? Many streptococcal infections disproportionately affect the very young and the very old – and this pattern is mirrored in the quality of the inflammasome response. Neonates and newborns have a diminished ability to produce inflammatory cytokines, such as IL-1β (143). Several mechanisms are at play, including immune system immaturity (144) and active suppression of innate immunity (145), and future work is required to better define the role of the inflammasome in these processes. A different mechanism may be at play in older populations, wherein TLR expression deficiency has been reported to mute cytokine activation in aged mice (146). Local lymphoid tissue responses are aberrant in aged mice, with baseline inflammation and high IL-1β levels already present in the lymphoid tissue of the upper respiratory tract in naive elderly mice, which then failed to upregulate NLRP3 and IL-1β in response to SPN colonization (147). Host genetics also plays a role – MyD88 and IRAK-4 are important for the IL–1β/inflammasome response, and mutations in these genes lead to susceptibility to pyogenic infections similar to those caused by the streptococci (148, 149). Other underlying conditions associated with severe streptococcal infections are inflammatory diseases including diabetes and super-infection by other pathogens, either of which can alter inflammasome responses.

Can pharmacologic targeting of the inflammasome provide a therapeutic benefit during streptococcal infection? Knockout mice deficient in inflammasome factors or inflammasome-regulated cytokines are generally more susceptible to experimental infection. Restoration with exogenous IL-1β is protective in models of GBS septicemia (5) and SPN nasopharyngeal colonization (9, 20). Exogenous IL-18 was also protective in models of GBS sepsis and neonatal infection (39). SPN isolates that do not induce hemolysis or inflammasome activation induce less IL-1β and cause more invasive disease (30, 55, 150). Correspondingly, PLY-mutant SPN bacteria that induce less IL-1 and inflammasome activation (81) are better able to establish chronic infection (151). This mechanism of “flying under the radar” by avoiding inflammasome activation, even at the consequence of losing an important virulence factor, is becoming a paradigm in the field of bacterial pathogenesis. Future therapeutics that take into account the inflammasome pathway when targeting bacterial pathogens may hold promise for better outcomes in treatment of serious bacterial infections.

## Conflict of Interest Statement

The authors declare that the research was conducted in the absence of any commercial or financial relationships that could be construed as a potential conflict of interest.
